# Longitudinally transverse myelitis as a rare complication of pneumococcal meningitis: case report

**DOI:** 10.11604/pamj.2024.49.11.43180

**Published:** 2024-09-09

**Authors:** Hela Sakly, Sana Rouis, Hajer Chakroun

**Affiliations:** 1 Infectious Diseases Department, Ibn Al Jazzar University Hospital, Kairouan, Tunisia,; 2 Faculty of Medicine of Sousse, University of Sousse, Sousse, Tunisia

**Keywords:** Bacterial meningitis, pneumococcal, infectious myelitis, case report

## Abstract

Pneumococcal meningitis is the most severe bacterial meningitis rarely complicated by acute myelitis. We report a case of a 54-year-old female who presented with pneumococcal meningoencephalitis. After eight days of hospitalization, the patient presented a sudden onset of bilateral lower leg weakness and bladder and bowel sphincter dysfunction. A neurological examination revealed a flaccid paraplegia. A magnetic resonance imaging (MRI) of the brain and the complete spinal cord was performed and showed cervical and thoracic myelitis. The patient was successfully treated with antibiotics and intravenous steroids. This case emphasizes the importance of considering myelitis as an acute complication of bacterial pneumococcal meningitis, with a good prognosis depending on early recognition and administration of appropriate therapy.

## Introduction

Bacterial meningitis is a medical emergency requiring immediate diagnosis and treatment. *Streptococcus pneumoniae* and *Neisseria meningitidis* are the most common and aggressive pathogens of meningitis [1]. Pneumococcal meningitis is the most severe bacterial meningitis and has the worst clinical prognosis. Survival depends on early recognition, followed by administration of appropriate antibiotic therapy. Neurological complications of community-acquired pneumococcal meningitis such as edema, vascular alterations and hydrocephalus are rare but can be observed [2]. Longitudinally extensive transverse myelitis (TM) is an unusual complication with few cases reported in the literature [3]. We present a case of an adult patient diagnosed with acute pneumococcal myelitis.

## Patient and observation

**Patient information:** a 54-year-old female presented to the emergency department (ED) with a four-day history of fever, headache, and vomiting. She had a previous history of two anterior episodes of pneumococcal meningitis treated with antibiotics with a good clinical outcome.

**Clinical findings:** the initial examination in the ED revealed fever, Glasgow Coma Scale Score (GCS) at 8, neck stiffness, and no focal neurological deficit.

### Timeline of current episode

**Diagnostic assessment:** laboratory analysis revealed hyperleukocytosis at 14300 cells/mm^3^, and anemia at 10 g/dl. The cerebrospinal fluid was trouble with pleocytosis at 360 cells/mm^3^ (85% granulocytes and 15% lymphocytes). The protein level was about 1.6 g/l. The cerebrospinal fluid level of glucose per the blood level of glucose was less than 0.5 (0.08). The brain CT scan was normal. The cerebrospinal fluid culture revealed cefotaxime and penicillin-sensitive *Streptococcus pneumoniae* (Annex 1).

**Figure 1:**
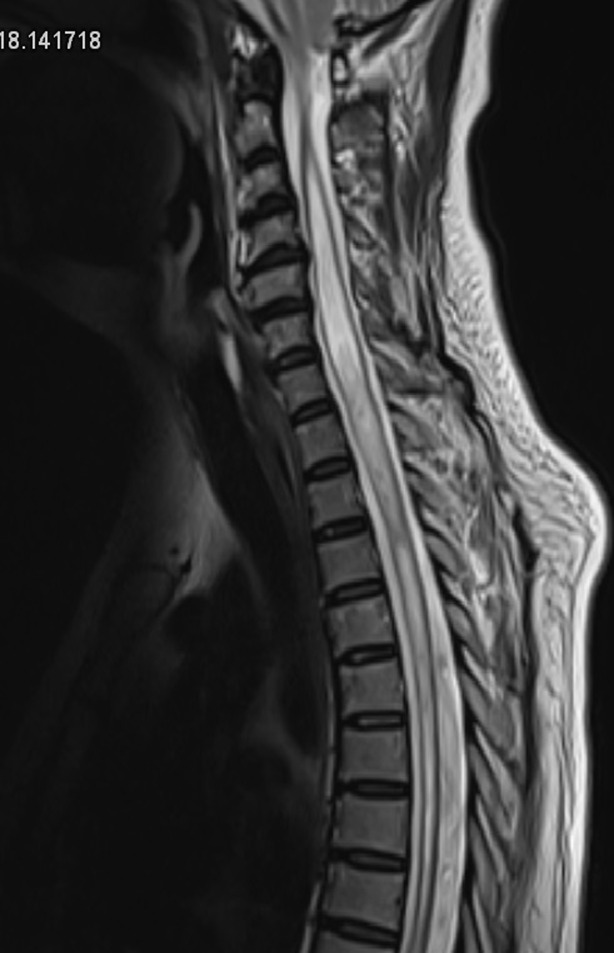
central intramedullary hyperintensities ranging from C3 to T11

**Diagnosis:** the patient was admitted to the intensive care unit. In front of the worsening of his neurological, she was promptly intubated and put on assisted invasive mechanical ventilation. The prescribed treatment was intravenous cefotaxime 300 mg/kg/day and dexamethasone 40 mg per day for 4 days. On day four of antibiotics, the patient had an important clinical improvement and was successfully weaned from the ventilator and was transferred to the infectious diseases department. On day eight of hospitalization, the patient presented difficulty in walking and standing, progressive paresis, bowel and bladder sphincter dysfunction with constipation and urine retention that required a urethral catheter and resumption of fever. The neurological examination revealed flaccid paraplegia and muscle strength of 2 in her lower extremities. Sensibility and vibratory sense were decreased in both legs and anal tone was normal. A magnetic resonance imaging (MRI) of the brain and the complete spinal cord was performed for suspected myelitis. The MRI showed long segment T2 extensive intramedullary hyperintensity causing cord expansion from C3 to T11 level and heterogeneous central cord hyperintensity (Figure 1). Brain and bilateral optic nerves were normal. Viral serologies (EBV, CMV, Parvovirus B19 and West-Nile) and immunological investigations (antinuclear antibodies (ANA), rheumatoid factor, antiphospholipid antibodies (APL), anti-neutrophil cytoplasmic antibodies (ANCA), anti-aquaporine 4, anti-MOG (anti-myelin-oligodendrocyte glycoprotein) were performed. No viral cause or immunological disorder was found. Neuro-ophtalmological evaluation was normal. The diagnosis of longitudinally extensive transverse pneumococcal myelitis was confirmed.

**Therapeutic interventions:** the patient was given high-dose intravenous methylprednisolone 1g per day for 20 days with a progressive decrease in doses and switched to oral corticosteroid for 2 months.

**Follow-up and outcome of interventions:** the outcome was good, and the patient had a moderate improvement in muscle strength and walking. She was discharged from the hospital with an indwelling silicone urinary catheter and was referred to a rehabilitation department. After four months of follow-up, the patient had improved her muscle strength. After urinary catheter removal, the outcome was good and there was no urinary retention. On follow-up, the patient underwent an MRI of the complete spinal cord which indicated that abnormalities had been resolved within two months of corticosteroids (lost MRI images from the file and the radiology department).

**Patient perspective:** the patient and his family were satisfied with the health care we provided during the hospitalization, and they were hopeful about his health outcomes.

**Informed consent:** the patient was informed about the publication of the case report and why the authors wanted to report it. A verbal consent was obtained from the patient for the clinical data to be published in the journal.

## Discussion

Acute bacterial meningitis can rarely cause polyradiculopathy and severe damage to the peripheral nerves. Transverse myelitis (TM) constitutes a rare syndrome with significant neurologic implications and requires urgent attention. This complication is rare in adults and has been reported in children [3]. Several pathophysiologic mechanisms such as inflammation, thrombosis and alteration of central nervous system metabolism have been proposed to explain spinal cord infarction in the presence of meningeal infection [4]. Adhesive arachnoiditis can occur from 10 days to several years after acute meningitis.

In our case, myelitis was suspected because of the sudden onset of lower extremities weakness, flaccid paraplegia, and bladder sphincter dysfunction. MRI of the spinal cord is the first mandatory exam to explore patients with myelopathic features and exclude urgent complications such as spinal cord compression [5,6]. The extensive central intramedullary hyperintensities on T2 MRI, the low intramedullary signal on T1 weighted imaging and the discrete nodular contrasts enhancement were primarily compatible with myelitis (Figure 1). A variety of etiologies can cause myelitis such as systemic autoimmune disorders and acquired demyelinating diseases [4]. Systemic inflammatory autoimmune disorders (such as systemic lupus erythematosus (SLE), Sjögren syndrome (SS), neurosarcoidosis or neuro-Behcet’s disease), acquired demyelinating disorders, viral, bacterial, and fungal [4,7] agents can lead to transverse myelitis [4]. Viral serologies (VS), rheumatoid factor, (RS), and anti-neutrophil cytoplasmic antibodies (ANCA) must be obtained when other cause of myelitis is clinically suspected [8].

As early as possible, a high intravenous dose of methylprednisolone (1g daily for 3 to 7 days) should be prescribed to relieve symptoms and improve the outcome [9]. Intravenous steroid is often instituted for patients with an acute phase of transverse myelitis. Though, there is no randomized, placebo-controlled study, to support this treatment [9]. A study of five children with severe TM after bacterial meningitis who received Solumedrol (1g/1.73 meter squared per day) for 3 or 5 days followed by oral prednisone for 14 days reported beneficial effects compared to ten historic controls [7,10]. In the steroid-treated group, the median time to walking was 23 days vs 97 days, full recovery occurred in 80% vs 10%, and full motor recovery at 1 year was present in 100% vs 20%. Plasma exchange and immune-modulator therapies are indicated for patients with chronic immune disorders [10].

Long-term management of myelitis consists of rehabilitative care to prevent secondary complications of immobility and improve their functional skills. In our case, the outcome was good with corticosteroids. The patient improved her muscle strength in the lower extremities within four months of the initial consultation. On follow-up, she underwent an MRI of the complete spinal cord. Signal abnormalities have resolved within two months of corticosteroids. Infectious myelitis has often a better prognosis than myelitis associated with auto-immune disorders [7]. The nerve damage caused by immunological mechanisms can be irreversible when diagnosed at a late stage of the disease. Thus, as demonstrated in this case, myelitis should be considered as an acute complication of bacterial pneumococcal meningitis, with a good prognosis when diagnosed and treated at an early stage. Here, we report the first case of pneumococcal acute myelitis due to *Streptococcus pneumoniae* in Tunisia.

## Conclusion

We report a rare case of acute pneumococcal meningitis complicated by myelitis treated with steroids and a good clinical improvement. Physicians should be able to early recognize this complication with a daily neurological examination. MRI is the best exam to diagnose myelitis. Steroids are the treatment of choice. Although rare, prompt management and regular follow-up will prevent significant morbidity with a good prognosis.

## Annex

**Annex 1**: antimicrobial susceptibility testing of *Streptococcus pneumonia* isolated in the cerebrospinal fluid (303 KB)
